# Sufficient sleep and its contributing factors among high school students during the COVID-19 pandemic: results from adolescent behaviors and experiences survey

**DOI:** 10.3389/fpubh.2024.1408746

**Published:** 2024-08-16

**Authors:** Zhengyang Chen, Ruili Li, Yuexi Liu, Qiguo Lian

**Affiliations:** ^1^Preventive Care Department, Jiangyou Fifth People’s Hospital, Sichuan, China; ^2^Children Health and Development Department, Capital Institute of Pediatrics, Beijing, China; ^3^The Second Clinical Medical College, Dalian Medical University, Dalian, China; ^4^NHC Key Lab of Reproduction Regulation, Shanghai Institute for Biomedical and Pharmaceutical Technologies, Shanghai, China

**Keywords:** sufficient sleep, COVID-19, adolescents, adolescent behaviors and experiences survey, mental health

## Abstract

**Background:**

The COVID-19 pandemic has caused profound changes in adolescent lives, including school closures, social isolation, family economic hardship, and sleep schedule. We aimed to assess the risk and protective factors of sufficient sleep among adolescents during COVID-19.

**Methods:**

We conducted secondary analysis based on the cross-sectional school-based Adolescent Behaviors and Experiences Survey in 2021 (*n* = 7,705). The ABES collected information on health-related experiences and behaviors during COVID-19. The outcome was sufficient sleep (eight and more hours of sleep on the average school night). The contributing factors included demographic, mental health, and adverse experiences indicators. We estimated the prevalence of sufficient sleep within each factor, and examined their associations using Chi-square test. We further investigated the contributing factors of sufficient sleep using multivariate logistic regression and reported the adjusted odds ratios (AORs) and 95% confidence intervals (CIs).

**Results:**

During January–June 2021, 23.5% of the U.S. high school students reported getting sufficient sleep. The multivariate logistic regression indicated that younger age (AOR, 2.04; 95%CI, 1.59–2.62), heterosexual identity (AOR, 1.61; 95%CI, 1.19–2.18), no poor mental health during the past 30 days (AOR, 1.37; 95%CI, 1.03–1.82), no persistent feelings of sadness or hopelessness (AOR, 1.34; 95%CI, 1.09–1.66), no food and nutrition insecurity (AOR, 1.47; 95%CI, 1.17–1.85), never been abused by a parent emotionally (AOR, 1.38; 95%CI, 1.16–1.64), and no schoolwork difficulty (AOR, 1.24; 95%CI, 1.01–1.51) were associated with sufficient sleep.

**Conclusion:**

We estimated the national prevalence of adolescent sufficient sleep during the COVID-19 pandemic and found that younger students, sexual heterosexual students, and students without certain mental health conditions or adverse experiences are at higher likelihood of sufficient sleep. These findings can help develop effective interventions on sleep duration in the response to a possible future pandemic caused by Disease X.

## Introduction

The COVID-19 pandemic has significantly impacted adolescent health worldwide in multifaceted ways, and the impact can be both negative and positive (1). Adolescents have unique developmental needs during the pandemic. The vast majority of research has been on the negative impacts of the COVID-19 pandemic. As a result of school closures, social distancing and the interruption of study are especially challenging for adolescent students (1, 2). In addition to depression and suicide concerns and psychosocial adjustments (3–5), adolescents are also venerable to domestic violence (5) and abuse (6).

Sleep is a core behavior of human being, especially for adolescents. Insufficient sleep poses a significant and multifaceted set of health risks in adolescents, including school performance, mental health and brain structure (7, 8). The positives of the COVID-19 pandemic for adolescents include improvements in family support and increases in sleep duration (9, 10). However, exiting studies, including cross-sectional and longitudinal design (sample size ranged from 590 to 8,972), on the changes in sleep duration among adolescents during the pandemic have led to mixed results (10–14). For example, although a few studies demonstrated that adolescents usually reported longer sleep duration during school closures (10–13), a longitudinal study found a significant decrease in sleep duration among Chinese adolescents (14).

It is also important to note that many adolescents also experienced factors that might increase the risk of short sleep duration during the pandemic, including mental health consequences (15–17), family financial insecurities (18), and child abuse and neglect (18). However, to date, little is known about the protective factors contributing to adolescent sleep duration on a personal level during the pandemic.

The cumulative COVID-19 cases and deaths in USA surged from 20,271,441 and 362,570 on January 3, 2021 to 85,970,435 and 1,007,412 on June 26, 2021, respectively, according to the World Health Organization COVID-19 dashboard data.^1^ To better understand the impact of COVID-19 on adolescent sleep duration and identify major protective factors, we used the Adolescent Behaviors and Experiences Survey (ABES) data during January–June 2021 to examine the prevalence of sufficient sleep and associated risk and protective factors among U.S. high school students. The findings in this study suggest developing programs that can increase the sleep duration of all students during and after the pandemic. We, based on existing literature, hypothesized that adolescents identified as heterosexual, without mental health conditions or adverse experiences exposure were more likely to reported eight or more hours of sleep per night.

## Materials and methods

### Study design and participants

This study includes data from the ABES, a one-time, representative online survey of U.S. high school students conducted by the U.S. Centers for Disease Control and Prevention (CDC) from January–June 2021 to assess student experiences during the COVID-19 pandemic. ABES employed a stratified, three-stage cluster sample to obtain the U.S. nationally representative sample of high school students in grades 9–12. There were 7,705 students from 128 participating schools. Participation in ABES was voluntary, and parental permission was granted. In ABES, the response rate was 38% at the school level, and 48% at the student level, resulting in an overall response rate of 18% (19). This study used pre-existing de-identified and publicly available data; hence no further ethical approval was required. All data used in this study are available at https://www.cdc.gov/healthyyouth/data/abes.htm.

### Inclusion and exclusion criteria

All students a selected class in public and private schools with grades 9–12 chosen by ABES sampling frame were eligible for this study. Exclusion criteria included students whose parental permission was not granted, who did not agree to participate, and who were unable to complete the questionnaire independently (19).

### Measures

ABES was a 110-question survey, included 97 items from the 2021 national the national Youth Risk Behavior Survey (YRBS) questionnaire, 12 new items assessing COVID-19–related behaviors and experiences and one new question on perceived racism (19).

The primary outcome in this study is sufficient sleep. ABES measured the sleep time using a single item (19): “On an average school night, how many hours of sleep do you get?” with options 1 = 4 or less hours, 2 = 5 h, 3 = 6 h, 4 = 7 h, 5 = 8 h, 6 = 9 h, 7 = 10 or more hours. We identified sufficient sleep as self-reported eight or more hours of sleep on an average school night, according to the recommendation from the American Academy of Sleep Medicine (20).

This study also included other measures in ABES: (1) demographic characteristics, (2) mental health conditions, and (3) student experiences. Demographic variables included sex, grade, sexual identity, and school instructional model. Mental health variables included virtual connection with others, poor mental health during the pandemic, poor mental health during the past 30 days, persistent feelings of sadness or hopelessness during the past 12 months, serious consideration of attempting suicide during the past year, attempted suicide during the past year, feeling close to persons at school and being virtually connected to others during the pandemic (15). And student experiences variables during the COVID-19 pandemic included parental economic, food and nutrition, abuse by a parent or other adult in the home, and difficulty completing schoolwork (18). The ABES questionnaire items and analytic coding used in this study can be found in Table 1.

**Table 1:** Variables, questionnaire items, and analytic coding.

Variable	Questionnaire item	Analytic coding
**Outcome**		
Sufficient sleep	On an average school night, how many hours of sleep do you get?	Yes (≥8 h) versus no (<8 h)
**Demographic variable**		
Sex	What is your sex?	Female versus male
Grade	In what grade are you?	9th, 10th, 11th, 12th
Sexual identity	Which of the following best describes you?	Heterosexual, gay/lesbian/bisexual, other/questioning
**Mental health condition**		
Poor mental health during the pandemic	During the COVID-19 pandemic, how often was your mental health not good? (Poor mental health includes stress, anxiety, and depression)	Yes (always or most of the time) versus no (never, rarely, or sometimes)
Poor mental health during the past 30 days	During the past 30 days, how often was your mental health not good? (Poor mental health includes stress, anxiety, and depression)	Yes (always or most of the time) versus no (never, rarely, or sometimes)
Persistent feelings of sadness or hopelessness	During the past 12 months, did you ever feel so sad or hopeless almost every day for 2 weeks or more in a row that you stopped doing some usual activities?	Yes versus no
Seriously considered attempting suicide	During the past 12 months, did you ever seriously consider attempting suicide?	Yes versus no
Attempted suicide	During the past 12 months, how many times did you actually attempt suicide?	≥1 time versus 0 times
Felt close to persons at school	Do you agree or disagree that you feel close to people at your school?	Strongly agree or agree versus not sure, disagree, or strongly disagree
**Student experience during the pandemic**		
Parental job loss	During the COVID-19 pandemic, did a parent or other adult in your home lose their job even for a short amount of time?	Yes versus no
Food and nutrition insecurity	During the COVID-19 pandemic, how often did you go hungry because there was not enough food in your home?	Yes (rarely, sometimes, most of the time, or always) versus no (never)
Emotional abuse by a parent	During the COVID-19 pandemic, how often did a parent or other adult in your home swear at you, insult you, or put you down?	Yes (rarely, sometimes, most of the time, or always) versus no (never)
Physical abuse by a parent	During the COVID-19 pandemic, how often did a parent or other adult in your home hit, beat, kick, or physically hurt you in any way?	Yes (rarely, sometimes, most of the time, or always) versus no (never)
Schoolwork difficulty	Do you agree or disagree that doing your schoolwork was more difficult during the COVID-19 pandemic than before the pandemic started?	Yes (strongly agree, or agree) versus no (not sure, disagree, or strongly disagree)
Virtually connected to others	During the COVID-19 pandemic, how often were you able to spend time with family, friends, or other groups, such as clubs or religious groups, by using a computer, phone, or other device? (Do not count attending school online)	Yes (sometimes, most of the time, always) versus no (never or rarely)

### Statistical analysis

We conducted all analyses using Stata/SE (version 15.1; StataCorp LLC) to account for the complex survey design of the ABES, including primary sampling units, sampling strata, and overall analysis weight (19). We first estimated weighted prevalence and 95% confidence intervals (CIs) of sufficient sleep using Taylor series linearization by demographic characteristics, mental health conditions, and selected student experiences. Next, we used Chi-square tests to examine the bivariate associations of sufficient sleep with other measures. Furthermore, we adopted multivariate logistic regression to investigate the contributing factors of sufficient sleep after controlling the covariates listed in Table 2, and reported the adjusted odds ratios (AORs) and 95% confidence intervals (CIs). Statistical tests were considered to be significant if two-tailed *p* < 0.05. Complete case analyses were used.

**Table 2:** Student characteristics in adolescent behaviors and experiences survey.

Variable	Unweighted N	%^†^ (95% CI)
**Sufficient sleep**		
Yes	1,644	23.5 (21.6–25.7)
No	5,430	76.5 (74.3–78.4)
**Demographic variable**		
**Sex**		
Female	3,999	50.4 (46.9–53.9)
Male	3,678	49.6 (46.1–53.1)
**Grade**		
9	2,144	26.7 (24.0–29.6)
10	1,949	25.5 (23.1–28.0)
11	1,858	24.3 (2.23–26.4)
12	1,731	23.6 (21.2–26.1)
**Sexual identity**		
Heterosexual	5,539	77.5 (75.6–79.4)
Gay, lesbian, or bisexual	977	13.2 (11.9–14.6)
Other or questioning	648	9.3 (8.3–10.4)
**Mental health condition**		
**Poor mental health during the pandemic**		
Yes (always or most of the time)	2,643	37.1 (34.6–39.6)
No (never, rarely, or sometimes)	4,564	62.9 (60.4–65.4)
**Poor mental health during the past 30 days**		
Yes (always or most of the time)	2,165	31.1 (28.5–33.7)
No (never, rarely, or sometimes)	4,880	68.9 (66.3–71.5)
**Persistent feelings of sadness or hopelessness**		
Yes	3,359	44.2 (41.6–46.8)
No	4,303	55.8 (53.2–58.4)
**Seriously considered attempting suicide**		
Yes	1,538	19.9 (18.0–22.0)
No	6,090	80.1 (78.0–82.0)
**Attempted suicide**		
Yes (≥1 time)	628	9.0 (7.7–10.5)
No (0 times)	6,110	91.0 (89.5–92.3)
**Felt close to persons at school**		
Yes	3,279	46.6 (44.1–49.2)
No	3,767	53.4 (50.8–55.9)
**Student experience during the pandemic**		
**Parental job loss**		
Yes	1,922	28.5 (26.2–30.9)
No	4,963	71.5 (69.1–73.8)
**Food and nutrition insecurity**		
Yes (rarely, sometimes, most of the time, or always)	1,698	23.8 (21.6–26.3)
No (never)	5,483	76.2 (73.7–78.4)
**Emotional abuse by a parent**		
Yes (rarely, sometimes, most of the time, or always)	3,957	55.1 (52.3–57.8)
No (never)	3,176	44.9 (42.2–47.7)
**Physical abuse by a parent**		
Yes (rarely, sometimes, most of the time, or always)	876	11.3 (10.2–12.4)
No (never)	6,264	88.7 (87.6–89.8)
**Schoolwork difficulty**		
Yes (strongly agree, or agree)	4,826	66.6 (64.5–68.6)
No (not sure, disagree, or strongly disagree)	2,345	33.4 (31.4–35.5)
**Virtually connected to others**		
Yes (always, most of the time, or sometimes)	5,005	71.8 (70.2–73.3)
No (never or rarely)	2,080	28.2 (26.7–29.8)

CI, confidence interval. ^†^ Estimates are weighted.

## Results

The weighted percentage of students in the sample was 50.4% (95%CI, 46.9–53.9%) female and 49.6% (46.1–53.1%) male. Grade levels were 26.7% (95%CI, 24.0–29.6%) in 9th grade, 25.5% (23.1–28.0%) in 10th grade, 24.3% (2.23–26.4%) in 11th grade, and 23.6% (21.2–26.1%) in 12th grade. For sexual identity, 77.5% (95%CI, 75.6–79.4%) of students identified as heterosexual, 13.2% (11.9–14.6%) identified as gay, lesbian, or bisexual, 9.3% (8.3–10.4%) identified as other or questioning. There were 23.5% (95%CI, 21.6–25.7%) of high school students who reported getting sufficient sleep on an average school night (Table 2).

In univariate analyses (Table 3), male students reported a higher prevalence of sufficient sleep than female students (25.7% versus 21.7%). The prevalence of sufficient sleep was higher among younger students (grade 9, 28.0%; grade 12, 18.1%). Sufficient sleep was more prevalent in heterosexual students (25.3%) than in gay/lesbian/bisexual (14.2%) and other/questioning (17.8%) students (Table 3). However, the prevalence of sufficient sleep ranged from 22.2 to 27.3% in the 3 instructional models of school, and there was a non-significant difference in the prevalence across the 3 models (*p* = 0.096).

**Table 3:** Percentage of sufficient sleep by selected demographic characteristics, mental health conditions and student experiences.

Variable	%^†^ (95% CI)	*p* value^§^
**Demographic characteristics**		
**Sex**		
Female	21.7 (19.5–24.2)	0.009
Male	25.7 (23.1–28.5)	
**Grade**		
9	28.0 (24.9–31.3)	<0.001^***^
10	25.8 (22.5–29.4)	
11	21.8 (18.8–25.1)	
12	18.1 (15.1–21.5)	
**Sexual identity**		
Heterosexual	25.3 (23.0–27.8)	<0.001^***^
Gay, lesbian, or bisexual	14.2 (11.2–17.9)	
Other or questioning	17.8 (14.8–21.3)	
**Instructional model of school**		
In-person only	26.6 (18.4–36.8)	0.096
Virtual only	27.3 (22.1–33.1)	
Hybrid	22.2 (20.5–24.1)	
**Mental health condition**		
**Poor mental health during the pandemic**		
Yes (always or most of the time)	15.5 (13.6–17.5)	<0.001^***^
No (never, rarely, or sometimes)	28.4 (25.7–31.2)	
**Poor mental health during the past 30 days**		
Yes (always or most of the time)	15.5 (13.6–17.5)	<0.001^***^
No (never, rarely, or sometimes)	28.4 (25.7–31.2)	
**Persistent feelings of sadness or hopelessness**		
Yes	15.9 (14.3–17.7)	<0.001^***^
No	29.7 (26.9–32.7)	
**Seriously considered attempting suicide**		
Yes	13.9 (11.8–16.2)	<0.001^***^
No	26.1 (23.8–28.4)	
**Attempted suicide**		
Yes (≥1 time)	12.4 (9.3–16.3)	<0.001^***^
No (0 times)	24.2 (22.1–26.5)	
**Felt close to persons at school**		
Yes (Strongly agree or agree)	25.9 (23.3–28.7)	0.001^**^
No (Not sure, disagree, or strongly disagree)	21.4 (19.2–23.7)	
**Student experience during the pandemic**		
**Parental job loss**		
Yes	20.9 (17.9–24.2)	0.039^*^
No	24.3 (22.1–26.5)	
**Food and nutrition insecurity**		
Yes (rarely, sometimes, most of the time, or always)	15.9 (13.8–18.2)	<0.001^***^
No (never)	25.9 (23.6–28.3)	
**Emotional abuse by a parent**		
Yes (rarely, sometimes, most of the time, or always)	17.8 (15.9–19.8)	<0.001^***^
No (never)	30.7 (27.8–33.7)	
**Physical abuse by a parent**		
Yes (rarely, sometimes, most of the time, or always)	15.9 (12.5–19.9)	<0.001^***^
No (never)	24.5 (22.3–26.8)	
**Schoolwork difficulty**		
Yes (strongly agree, or agree)	20.9 (18.8–23.2)	<0.001^***^
No (not sure, disagree, or strongly disagree)	28.9 (25.9–32.1)	
**Virtually connected to others**		
Yes (always, most of the time, or sometimes)	24.2 (21.9–26.7)	0.087
No (never or rarely)	21.6 (19.1–24.3)	

CI, confidence interval; NA, not applicable.

^†^ Estimates are weighted. ^§^ Chi-square test. ^*^p < 0.05, ^**^p < 0.01, ^***^ *p* < 0.001.

The prevalence of sufficient sleep varied by mental health conditions (Table 3). For example, sufficient sleep was more prevalent among students without poor mental health during the pandemic (28.4% versus 15.5%, *p* < 0.001) or during the past 30 days (28.4% versus 15.5%, *p* < 0.001), those without persistent feelings of sadness or hopelessness (29.7% versus 15.9%, *p* < 0.001), and those without suicidal ideation (26.1% versus 13.9%, *p* < 0.001) or attempts (24.2% versus 12.4%, *p* < 0.001). In addition, students who felt close to persons at school reported a higher prevalence of sufficient sleep than those who did not feel close to persons at school (25.9% versus 21.4%, *p* = 0.001).

Sufficient sleep differed by adverse experiences during the pandemic (Table 3). The prevalence of sufficient sleep was higher among students who did not experience parental job loss (24.3% versus 20.9%) or food and nutrition insecurity (25.9% versus 15.9%). Sufficient sleep was also more prevalent among students who were never abused by a parent emotionally (30.7% versus 17.8%) or physically (24.5% versus 15.9%). In addition, students who did not have difficulty completing their schoolwork reported a higher prevalence of sufficient sleep than those who reported difficulty completing their schoolwork (28.9% versus 20.9%). However, students who reported virtually connected to others had a similar prevalence of sufficient sleep to those who never or rarely connected to others (24.2% versus 21.6%, *p* = 0.087). Multivariate analyses showed that younger age (AOR, 2.04; 95%CI, 1.59–2.62) and heterosexual identity (AOR, 1.61; 95%CI, 1.19–2.18) were among demographic characteristics associated with sufficient sleep. Among mental health conditions, no poor mental health during the past 30 days (AOR, 1.37; 95%CI, 1.03–1.82) and no persistent feelings of sadness or hopelessness (AOR, 1.34; 95%CI, 1.09–1.66) were associated with sufficient sleep. Among student experiences during the pandemic, no food and nutrition insecurity (AOR, 1.47; 95%CI, 1.17–1.85), never been abused by a parent emotionally (AOR, 1.38; 95%CI, 1.16–1.64), and having no difficulty completing schoolwork (AOR, 1.24; 95%CI, 1.01–1.51) were associated with sufficient sleep (Table 4).

**Table 4:** Associations of sufficient sleep with contributing factors.

Variable	AOR^†^ (95% CI)	*p* value^§^
**Demographic characteristic**		
**Sex (Ref: female)**		
Male	0.89 (0.73–1.09)	0.258
**Grade (Ref: grade 12)**		
9	2.04 (1.59–2.62)	<0.001^***^
10	1.68 (1.30–2.17)	<0.001^***^
11	1.43 (1.07–1.92)	0.016^*^
**Sexual identity (Ref: gay, lesbian, or bisexual)**		
Heterosexual	1.61 (1.19–2.18)	0.002^*^
Other or questioning	1.23 (0.78–1.94)	0.368
**Instructional model of school (Ref: In-person only)**		0.096
Virtual only	0.91 (0.43–1.92)	0.800
Hybrid	0.71 (0.35–1.46)	0.343
**Mental health condition**		
**Poor mental health during the pandemic (Ref: Yes)**		
No	1.16 (0.90–1.50)	0.245
**Poor mental health during the past 30 days (Ref: Yes)**		
No	1.37 (1.03–1.82)	0.031^*^
**Persistent feelings of sadness or hopelessness (Ref: Yes)**		
No	1.34 (1.09–1.66)	0.006^*^
**Seriously considered attempting suicide (Ref: Yes)**		
No	1.13 (0.86–1.48)	0.388
**Attempted suicide (Ref: Yes)**		
No	1.00 (0.61–1.64)	0.985
**Felt close to persons at school (Ref: Yes)**		
No	0.94 (0.80–1.12)	0.506
**Student experience during the pandemic**		
**Parental job loss (Ref: Yes)**		
No	1.06 (0.85–1.33)	0.584
**Food and nutrition insecurity (Ref: Yes)**		
No	1.47 (1.17–1.85)	0.001^*^
**Emotional abuse by a parent (Ref: Yes)**		
No	1.38 (1.16–1.64)	<0.001^***^
**Physical abuse by a parent (Ref: Yes)**		
No	1.18 (0.85–1.65)	0.323
**Schoolwork difficulty (Ref: Yes)**		
No	1.24 (1.01–1.51)	0.036^*^
**Virtually connected to others (Ref: Yes)**		
No	1.03 (0.83–1.29)	0.753

Ref, reference group; AOR, adjusted odds ratio (adjusted for other variables).

^†^ Estimates are weighted. ^§^ Multivariate logistic regression. ^*^p < 0.05, ^**^p < 0.01, ^***^p < 0.001.

## Discussion

In this study, based on a nationally representative American adolescent sample from ABES, we provided evidence of the prevalence of sufficient sleep and its contributing factors among high school students during the COVID-19 pandemic. We found only a quarter of high school students reported getting the recommended amount of sleep. We detected that younger students, students identified as heterosexual, and without certain mental health conditions or adverse experiences are more likely to report at higher likelihood of sufficient sleep, providing hints for further interventions in the response to a possible future pandemic caused by Disease X.

Insufficient sleep remained widespread during the COVID-19 pandemic, affecting 76.5% of students in USA (21) and 75.41% of students in Shandong, China (22). Similarly, in our study nearly only a quarter of high school students achieved recommended amounts of sleep on an average school night.

This study provides the first evidence for the effect of instructional models on sleep duration among adolescents during the pandemic. During the COVID-19 pandemic, although different instructional models (i.e., in-person only, virtual only, and hybrid) were used across the United States, the students reported a similar prevalence of sufficient sleep.

Before and during the COVID-19 pandemic, sexual minority (23, 24) is linked to sufficient sleep. Consistent with existing studies (10, 23, 24), we found that heterosexual students reported a higher prevalence of sufficient sleep, which indicated that policies need to address the discrimination and challenge social norms for adolescent sexual minority.

Older adolescents tend to report insufficient sleep due to academic pressure before the COVID-19 pandemic (25). Exiting studies suggested that younger children were associated with a higher risk of insufficient sleep during the COVID-19 pandemic duo to lacking of understanding of the COVID-19 (26). In a senior high school sample, we found an association of older age and insufficient sleep, which is partly caused by longer electronic media use and higher academic pressure among older adolescents.

Before and during the COVID-19 pandemic, adolescent mental health issues remain an international public health concern. Before the COVID-19 pandemic, one study based on YRBS 2017 indicated that adolescent insufficient sleep was associated with suicidal ideation (AOR = 1.35, 95% CI = 1.16–1.58). Another review article revealed the links between adolescent insufficient sleep and depression (27). During the COVID-19 pandemic, in line with the results of other study (28), we identified two mental health conditions measured in ABES that were associated with a higher risk of insufficient sleep among U.S. high school students. More concretely, students with poor mental health during the past 30 days and persistent feelings of sadness or hopelessness both reported a higher risk of insufficient sleep. Considering the potential mental health consequences of the ongoing pandemic for adolescents (29), our findings underscore the particular need to address adolescent mental health needs in the context of sufficient sleep. Although one longitudinal survey in Chinese college students (30) suggested that insufficient sleep predicts the development and persistence of suicidal ideation during the initial COVID-19 outbreak (February 3rd to 10th, 2020) and the remission period (March 24th to April 3rd, 2020), we did not observe this association in ABES. The inconsistent results might be multifactorial, including differences in research sample and government response.

During the COVID-19 pandemic, changes also occurred on the family level, including financial difficulty, food insecurity, and domestic abuse (31). Adolescent positive experiences were associated with sufficient sleep among U.S. high school students. More concretely, students without food and nutrition insecurity, emotional abuse, or schoolwork difficulty reported a higher likelihood of sufficient sleep. The COVID-19 pandemic related stress contributes to domestic violence in the context of students’ decreased contact with mandated reporters because of school closures (32). Financial and food insecurities induced by COVID-19 are linked to increased child abuse (32). The findings highlight the need for enhanced violence intervention strategies. In addition, schoolwork difficulty was associated with a higher risk of insufficient sleep but virtually connected to others was not. Compared with before the pandemic, students with insufficient sleep were more likely to report greater difficulty doing schoolwork during the pandemic (21). In line with previous study (33), our findings highlighted the important of virtually connected to others in extending sleep duration. At last, these experiences that contribute to insufficient sleep are interconnected (34), and a multi-level intervention approach to sufficient sleep is needed. The education authority and government policy-makers could frame and implement guidelines to improve sleep to augment mental health conditions and experiences in next pandemics.

## Limitations

The study has three specific limitations in addition to the general limitations for the ABES (e.g., including cross-sectional design, representativeness, social desirability bias, and low response rates) (19). First, the outcome was measured by asking about the sleep duration on an average school night. However, the COVID-19 pandemic has been sweeping across the United States and the world; hence the question can be understood within the context of the pandemic. Second, more robust measurements for both sleep duration (e.g., actigraphy) and contributors (e.g., assessment scales) are need. ABES adopt items from YRBS and measured sleep, mental health and experience by single questions, although YRBS items demonstrated good validity in measuring sleep duration (35) and suicidal ideation and attempts (36). Third, ABES did not measure sleep disturbances (e.g., snoring and insomnia) and sleep quality that largely influence the sleep duration. Last, ABES did not ask students about the period of instructional models in the schools they are attending, which might influence the findings.

## Conclusion

Sleep duration is a significant public health concern during the ongoing COVID-19 pandemic. The findings in this study indicate that in addition to sexual minority students, students with mental health conditions with adverse experiences during the pandemic are at higher risk of insufficient sleep. Therefore, comprehensive strategies are needed to improve sleep duration among adolescents in future pandemics.

## Data Availability

The original contributions presented in the study are included in the article/supplementary material, further inquiries can be directed to the corresponding author.

## Footnotes

^1^https://data.who.int/dashboards/covid19
